# Whole-body vibration administered during a 3-week in-hospital multidisciplinary body weight reduction program increases resting energy expenditure in obese adolescents, a randomized clinical trial

**DOI:** 10.3389/fendo.2025.1642437

**Published:** 2025-09-17

**Authors:** Sofia Tamini, Gabriella Tringali, Roberta De Micheli, Mario Bernardo-Filho, Danúbia da Cunha de Sá-Caputo, Alessandro Sartorio

**Affiliations:** ^1^ Istituto Auxologico Italiano, IRCCS, Experimental Laboratory for Auxo-endocrinological Research, Piancavallo-Verbania, Italy; ^2^ Laboratory of Mechanical Vibrations and Integrative Practices-LAVIMPI, Department of Biophysics and Biometry, Institute of Biology Roberto Alcântara Gomes and University Polyclinic Piquet Carneiro, Rio de Janeiro State University, Rio de Janeiro, Brazil

**Keywords:** obesity, pediatric obesity, whole-body vibration, mechanical vibration, adolescents

## Abstract

**Background:**

Pediatric obesity is a growing global health concern, and interventions aimed at increasing resting energy expenditure (REE) have gained attention as complementary strategies to dietary restriction. Whole-body vibration (WBV), an innovative exercise mimetic, may offer metabolic and functional benefits, particularly in populations with limited exercise tolerance. This study was a randomized clinical trial aimed to evaluate the effects of incorporating WBV into a 3-week in-hospital multidisciplinary body weight reduction program (BWRP) in male adolescents with obesity.

**Methods:**

Twenty-three male adolescents with obesity (mean age: 15.7 ± 1.3 years; mean body mass index (BMI): 38.5 ± 5.6 kg/m^2^) hospitalized for a 3-week BWRP were randomly assigned to a control group receiving the standard BWRP (subgroup A, n = 12) or an experimental group receiving BWRP plus WBV sessions (subgroup B, n = 11). Anthropometric and clinical parameters, REE, and lower limb muscle power, evaluated using the stair-climbing test, were assessed before and after the intervention.

**Results:**

Both subgroups demonstrated significant reductions in body weight and BMI. REE showed a significant Time × Group interaction (p < 0.01), indicating differential responses between the two intervention groups. WBV training significantly increased REE in subgroup B, from 2470.1 ± 249.6 kcal/day at baseline to 2733.0 ± 310.8 kcal/day post-intervention (Δ = +262.9 kcal, p < 0.001). In contrast, subgroup A showed no significant change in REE, with values decreasing from 2204.0 ± 307.4 kcal/day to 2020.8 ± 442.5 kcal/day (Δ = ˗183.2 kcal, p = ns). REE change was supported by significant Time × Group interaction in a two-way repeated measures ANOVA (p = 0.002). Furthermore, a significant post-intervention correlation emerged between REE and anaerobic power only in subgroup B.

**Conclusion:**

The addition of WBV to a structured BWRP significantly increased REE in obese adolescents, beyond the effects of the BWRP alone. This finding supports the use of WBV as a promising adjunct strategy to conventional interventions in pediatric obesity.

## 1 Introduction

Over the past decades, obesity has become a primary global health concern, with its prevalence rising steadily among adults, adolescents and children. (1). According to the Global Burden of Disease Study, from 1990 to 2022, obesity has dramatically increased both in men and in women worldwide, with a particularly alarming rise in children and adolescents (2).

Efforts to treat or prevent obesity have traditionally focused on reducing energy intake. More recently, increasing total daily energy expenditure (TEE) is gaining attention as a complementary approach (3).

TEE is the total amount of energy consumed by an individual in 24 hours and comprises three main components: resting energy expenditure (REE), diet-induced thermogenesis (DIT), and energy expenditure from physical activity (4, 5).

DIT is a relatively constant parameter among individuals and contributes marginally to TEE, with a maximum of 10%, whereas REE and physical activity are the predominant and more variable components (5, 6). REE, which accounts for 60–75% of TEE, represents the energy expended at rest to maintain vital body functions (7). In particular, REE is defined as the body’s energy expenditure at rest, when a person is awake, in a post-absorptive, thermoneutral state, and has not exercised for typically 12 hours (8, 9). Physical activity is the most variable parameter, as it is highly dependent on lifestyle and environmental factors (4, 10, 11).

Individuals with obesity, including children and adolescents, tend to engage in sedentary lifestyles, and consequently, physical activity is very limited (4, 12, 13). In these populations, REE becomes the dominant component of TEE (14).

Increasing REE could, therefore, represent a valuable strategy for managing excess body weight in the obese and overweight population, as it can help facilitate a negative energy balance and reduce fat mass (15). It has been demonstrated that a way to increase REE is resistance training, which promotes muscle mass gain and thus elevates metabolic rate at rest (16).

Whole-body vibration (WBV) has recently emerged as a promising alternative or adjunct to conventional exercise in systemic vibratory therapy. WBV is an innovative exercise mimetic that utilizes a vibrating platform to generate mechanical vibrations transmitted through the body, leading to various physiological responses that have contributed to the documented benefits following its use (17). During WBV, skeletal muscles rapidly contract and relax at a certain frequency, promoting muscle anabolism and enhancing the stretch reflex by activating muscle spindles (17, 18). As a form of mimetic exercise, WBV is particularly attractive for populations with limited exercise capacity, such as obese individuals or the elderly (17).

Mechanical vibration is transmitted to the entire body of the individual during WBV exercise stimulating mechanoreceptors found in the skin, muscles, and joints. This mechanotransduction (promoted by mechanoreceptors as Piezo 1 and Piezo 2) is related to organ development and homeostasis.

WBV training has been widely used in sports, physiotherapy, and rehabilitation, being an efficient, fast, noninvasive, and easy-to-use training tool that not only effectively increases muscle strength, power and improves body composition, but also rapidly increases energy expenditure (19–31). For instance, Da Silva et al. reported that resistance training involving WBV increased oxygen uptake and energy expenditure compared to the same exercise without WBV in active men (32). In their work, Da Silva et al. suggested that WBV programs could be combined with other physical exercises and with dietary changes to favor the achievement of a negative energy balance and to maintain or increase muscle mass (32). Similarly, Rittweger et al. found that mechanical vibration training increased the energy metabolic rate and ability to oxidize sugar during exercise, thus accelerating fat loss (33). More recently, Huang et al. reported that mechanical vibration training increased energy expenditure during low-intensity exercise and enhanced excess post-exercise oxygen consumption, thereby improving exercise intensity in well-trained male students. (34).

These findings suggest that WBV may represent an innovative, feasible, and effective exercise alternative with therapeutic potential in metabolic diseases to increase REE and overall TEE, especially in individuals unable or unwilling to engage in traditional forms of exercise (17). To establish a correct protocol for WBV exercise, it is necessary to consider the individual’s position, the type of vibrating platform, and parameters such as amplitude, frequency, and intensity (26).

Based on these premises, the present study aimed to evaluate whether adding a series of WBV to an in-hospital multidisciplinary body weight reduction program (BWRP) could increase REE in adolescents with obesity, thereby supporting increased TEE and contributing to weight management efforts.

## 2 Materials and methods

### 2.1 Study design

This study was conducted as a single-center randomized clinical trial designed to evaluate the effects of whole-body vibration (WBV) on resting energy expenditure (REE) and anaerobic performance in adolescents with obesity undergoing a multidisciplinary BWRP. The trial was registered at ClinicalTrials.gov (Identifier: NCT06800872).

Participants were randomly allocated in a 1:1 ratio to either the standard BWRP (control group) or BWRP plus WBV intervention (experimental group) using a computer-generated randomization sequence created via www.randomizer.org. Allocation was performed after initial screening and baseline assessments to ensure unbiased distribution.

Due to the nature of the intervention, blinding of participants and intervention providers was not feasible; however, outcome assessors were blinded to group assignment to minimize bias in measurement.

### 2.2 Study population

Adolescents with obesity, hospitalized at the Division of Auxology, Istituto Auxologico Italiano, Piancavallo-Verbania, Italy, for a 3-week multidisciplinary BWRP, were screened for eligibility. Inclusion criteria were: (1) male individuals, aged < 18 years; (2) a body mass index standard deviation score (BMI SDS) > 2, based on age- and sex-specific Italian growth references (35). The BMI SDS is a standardized measure expressing how a subject’s BMI compares to a reference population of the same age and sex, reported as the number of standard deviations away from the mean. A BMI SDS greater than 2 indicates obesity, reflecting a BMI value significantly above average for age and sex, according to Italian growth reference charts (35).

Exclusion criteria were: (1) secondary causes of obesity (e.g., Prader–Willi syndrome, steroid-induced or medication-induced obesity); (2) comorbid psychiatric, neurological, osteomuscular, or rheumatologic disorders that could hinder adherence to the in-hospital rehabilitation protocol; and (3) refusal by the adolescent or parent/legal guardian to provide written informed consent.

Eligible subjects were randomly assigned to two subgroups. Subgroup A underwent the standardized BWRP, which entailed energy restriction, physical and nutritional education and psychological counselling, and served as a control group. Subgroup B was subjected to the same BWRP with the addition of a series of WBV sessions during hospitalization.

The study protocol was approved by the Territorial Ethical Committee no. 5, Lombardy Region, Milan, Italy, on May 21^st^, 2024 (EC reference number: 200/24; research project code: 01C413). The purpose and objectives of the study were explained in detail to each subject and their parents, and written informed consent was obtained prior to the commencement of the study.

### 2.3 Body weight reduction program

The BWRP consisted of a 3-week multidisciplinary in-hospital (i.e., full-time staying in the hospital, including the night) metabolic rehabilitation, entailing an energy-restricted diet, physical exercise, psychological counselling, and nutritional education. Caloric intake was individualized by subtracting approximately 500 kcal from each participant’s measured REE. The diet consisted of approximately 21% proteins, 53% carbohydrates, and 26% lipids. The daily estimated water content was 1000 mL, while the sodium, potassium, and calcium contents were approximately 1560 mg, 3600 mg, and 900 mg, respectively. An extra water intake of at least 2000 mL/day was encouraged. Meals were served at fixed times: breakfast (07:30 AM), lunch (12:30 PM), and dinner (07:30 PM). Breakfast included milk or yogurt with cereals or biscuits; lunch was composed of pasta or rice (first course), lean meat/fish/eggs (second course), vegetables, and fruit; and dinner included soup or vegetable puree with cereals or rice (first course), cheese/ham/fish (second course), vegetables, and fruit.

The physical activity program consisted of five training sessions per week, including (i) Aerobic training: 1 hour of moderate-intensity floor and standing exercises (e.g., squats, step-ups, rope jumping, push-ups, lunges), supervised by a physical therapist, with heart rate monitored using a Polar RS400SD device (Polar Electro Oy, Kempele, Finland). The target heart rate was set at 60–80% of the age-predicted maximum (220–age).; (ii) Endurance activity: either 20–30 minutes of cycle ergometry at 60 W (determined via incremental test at admission), or outdoor walking (3–4 km), depending on individual capability and medical status.

Psychological counselling included two to three weekly sessions of individual or group therapy, performed by clinical psychologists, based on cognitive behavioral therapy strategies such as stimulus control procedures, problem-solving and stress management training, development of healthy eating habits, assertiveness and social-skills training, cognitive restructuring of negative maladaptive thoughts, and relapse prevention training. Additional sessions were conducted with the adolescents’ parents, when possible (one day per week), to improve motivation for lifestyle changes and interpersonal communication.

Furthermore, lectures on the problems and risks of obesity, motivational speeches, examples of healthy foods, food preparation workshops, and group discussions (with or without a supervisor) took place daily.

### 2.4 WBV exercise

Participants in Subgroup B performed 24 WBV sessions (2 sessions/day, 12 days) during hospitalization, in addition to the standard five weekly exercise sessions included in the BWRP. Each WBV sessions were performed using a vibrating platform generating vertical sinusoidal mechanical vibrations (Nevisys H1, RME, Ferrara, Italy) with the subject in a squat position, with a 110° knee flexion, vibration stimulation at a frequency of 30 Hz and peak-to-peak displacement of 3 mm for a time of 60 seconds followed by 30 seconds of rest. Each WBV session consisted of a sequence of mechanical vibration-rest cycles repeated 20 times. Across the 12 days of training, participants received approximately 360 minutes of WBV-specific activity in addition to the conventional exercise prescribed as part of the BWRP.

The acceleration peak of the mechanical vibration was 2.85 g, as assessed by a magnetic monoaxial accelerometer (Vibration Meter, Lutron VB-8200, Lutron Electronic Enterprise Co., LTD, Taipei, Taiwan).

### 2.5 Anthropometric and clinical measurements

A medical history was obtained, and a physical examination was performed at baseline and at the end of the BWRP. Stature and body mass (BM) were measured using a Harpenden stadiometer (Holtain Ltd., United Kingdom) and an electronic scale (Selus, Italy), respectively, with the subject wearing only light underwear. Body mass index (BMI) was calculated as BM (in kilograms) divided by stature (in meters) squared. The standard deviation score (SDS) of BMI was calculated using the LMS method (36).

Body composition was measured at the beginning and end of the BWRP using a multifrequency tetrapolar impedancemeter (Bioelectrical Impedance Analysis, BIA, Human-IM Scan, DS-Medigroup, Milan, Italy) with a delivered current of 800 mA at a frequency of 50 kHz. Care was taken to standardize the variables affecting the measurement’s validity, reproducibility and precision to reduce measurement errors.

The measurements were performed according to the method of Lukaski et al. (after 20 min of rest in the supine position with arms and legs relaxed and without contact with other parts of the body) and under strictly controlled conditions according to National Institutes of Health (NIH) guidelines (37, 38). Before measurements, technical accuracy has been validated by an external parallel circuit containing high-precision resistors and capacitors. Low-impedance electrodes were used for reliable and accurate assessment of the raw bioimpedance parameters (e.g., R, Xc, and phase angle) (38). The within-day coefficient of variation for three repeated assessments of FFM in obese subjects (with repositioning of electrodes) has been previously assessed in the laboratory (2.4%).

Fat-Free Mass (FFM) was calculated using the following prediction equation:


$$\text{FFM }(\text{kg})=0.87\;\times ZI(cm^{2}\text{ x }Ω)+3\times 1\;(\text{adjusted coefficient of variation}=0.91)$$

where *ZI* is the impedance index calculated as stature (cm^2^) divided by whole-body impedance (*Z*) at 50 kHz (Ω). Fat mass (FM, kg) was derived as the difference between BM (kg) and FFM (kg) (39).

Systolic (SBP) and diastolic blood pressure (DBP) were measured twice (3-minute intervals in between) on the dominant arm with an aneroid sphygmomanometer (TemaCertus, Milan, Italy), by using appropriate-sized cuffs for young participants with obesity. The mean values were calculated and rounded to the nearest 5 mmHg value.

Resting heart rate monitored using a Polar RS400SD device (Polar Electro Oy, Kempele, Finland).

### 2.6 REE measurement

REE was measured between 8:00 and 10:00 AM in thermoneutral conditions (room temperature: 22°-25 °C) using an open-circuit, indirect, computerized calorimeter equipped with a canopy (Vmax 29, Sensor Medics, Yorba Linda, CA). The calorimeter underwent periodic quality control tests to ensure the reliability of the measurements. The gas analyzers were calibrated before each test using a reference gas mixture of 15% O2 and 5% CO2. The participants were fasting for at least 8 hours, had not smoked for at least 1 hour, and waited 30 minutes in a sitting position before undergoing REE measurement. REE was assessed in the supine position for at least 30 minutes, including an acclimation period of 10 minutes. The data related to the acclimation period were discarded. The steady state was defined as at least 5 minutes with less than 5% variation in the respiratory quotient, ventilation (40). After the steady state was reached, O2 consumption and CO2 production were recorded at 1-minute intervals for at least 20 minutes and averaged over the entire measurement period. REE was calculated from O2 consumption and CO2 production using Weir’s equation (41).

### 2.7 Stair climbing test

The Stair Climbing Test (SCT) is a well-standardized procedure for measuring maximal anaerobic power in adolescents and adults with obesity. The patients were invited to climb ordinary stairs (13 steps, each 15.3 cm high, with a vertical distance of 1.99 m) at the highest possible speed, according to their capabilities. The SCT time (T_SCT_) taken to perform the test was measured with a digital stopwatch. The determination of the time starts when the first foot is elevated and finishes with the contact of the foot on the floor of the last step. Before the actual test, 2–3 practice trials were scheduled to allow subjects to gain sufficient confidence with the technique. SCT repeatability in obese subjects has been previously evaluated, and the coefficient of variation between measurements was found to be lower than 5% (42, 43).

Lower limb muscle power (P_SCT_) was calculated by the following equation:


$$P_{SCT}=\frac{bm\times h\times g}{T_{SCT}}$$

where *bm* represents body mass (expressed in kg), *h* is the stature (m) of the total vertical distance of the stairs, and *g* (m/s^2^) is the acceleration of gravity, which is 9.81 m/s^2^ (44, 45).

### 2.8 Statistical analysis

The sample size was calculated *a priori* based on expected differences in REE between groups. Assuming a mean REE of approximately 1950 kcal/day in adolescents with obesity, a clinically meaningful difference of 195 kcal/day between groups, and a standard deviation (σ) of 160 kcal, a total of 24 participants (12 per group) was required to achieve 80% power (1-β = 0.80) at a two-sided alpha level of 0.05. The calculation was performed using PASS 21 Power Analysis and Sample Size Software (2021) (NCSS, LLC. Kaysville, Utah, USA).

Continuous variables are expressed as mean ± standard deviation. The Shapiro-Wilk test was performed to verify that all parameters were normally distributed. The change (Δ) in the main outcome variables (e.g., REE, anaerobic power) was calculated as the difference between post- and pre-intervention values (Δ = post-pre).

Primary analyses were conducted using a two-way repeated measures ANOVA to examine the effects of time (pre vs. post) and group (Subgroup A vs Subgroup B) on all the variables. When the ANOVA showed a significant Time × Group interaction, Bonferroni-adjusted *post hoc Student’s t-tests* for paired and unpaired samples, as appropriate, were conducted to explore within-group (pre vs. post) and between-group (Subgroup A vs Subgroup B) differences.

Furthermore, to evaluate the relationship between REE and anaerobic power, as measured by P_SCT_, Pearson’s correlation was performed, and the r and r-squared (r2) values were calculated at both pre- and post-intervention time points. Pearson’s correlation assumptions were verified before the analysis. Correlation strength was interpreted according to the following thresholds: r < ± 0.10 indicates a negligible correlation; r between ± 0.11 and ± 0.39 a weak correlation, r between ± 0.40 and ± 0.69 a moderate correlation, r between ± 0.70 and ± 0.89 a strong correlation and r > ± 0.90 a very strong correlation (46). To compare correlation strength between groups, Fisher’s r-to-z transformation was applied at baseline and post-intervention.

A level of significance of *p* < 0.05 was used for all data analysis. All statistical analyses were performed using GraphPad Prism software (Version 10; GraphPad Software, San Diego, CA, USA).

## 3 Results

Twenty-four male adolescents with obesity, hospitalized at the Division of Auxology, Istituto Auxologico Italiano, Piancavallo-Verbania, Italy for a 3-week multidisciplinary BWRP, were initially recruited and randomly assigned to the two subgroups. However, one participant in the WBV group voluntarily withdrew due to early discharge from the hospital for personal reasons. Consequently, 23 participants (mean age: 15.7 ± 1.3 yrs; mean BMI: 38.5 ± 5.6 kg/m^2^) completed the study and were included in the final analysis. In particular, 12 participants were allocated to the control group (Subgroup A) and 11 to the WBV group (Subgroup B). Baseline characteristics did not differ significantly between groups.

The main anthropometric and clinical characteristics of the two subgroups are shown in **Table 1**. Detailed results of the two-way repeated measures ANOVA analysis are reported in **Supplementary Materials, Table 1**. Results of the post-hoc comparisons for significant time × group interactions (Bonferroni-corrected) are reported in **Table 2**.

**Table 1:** Main characteristics of the study population in the two subgroups at baseline and post the BWRP.

	Total	Subgroup A		Subgroup B		P value*
		PRE	POST	PRE	POST	
n.	23	12		11		–
Age (yrs)	15.7 ± 1.3	15.5 ± 1.5		15.9 ± 1.2		–
BM (kg)	117.3 ± 21.1	115.3 ± 25.5	110.2 ± 24.0	119.6 ± 15.8	115.3 ± 14.9	ns
Stature (cm)	174.2 ± 7.8	171.9 ± 8.4		176.7 ± 6.4		–
BMI (kg/m^2^)	38.5 ± 5.6	38.7 ± 6.1	37.0 ± 5.8	38.3 ± 5.2	36.9 ± 5.4	ns
BMI SDS	3.2 ± 0.7	3.2 ± 0.7	3.0 ± 0.7	3.1 ± 0.6	2.9 ± 0.6	ns
FFM (%)	58.4 ± 5.3	57.9 ± 5.1	59.6 ± 4.9	58.9 ± 5.8	60.3 ± 4.2	ns
FM (kg)	50.0 ± 13.2	49.4 ± 15.5	44.0 ± 13.2	50.0 ± 10.5	44.4 ± 8.6	ns
SBP (mmHg)	129.6 ± 10.8	128.3 ± 7.5	117.1 ± 10.1	130.9 ± 13.8	117.3 ± 6.5	ns
DBP (mmHg)	80.7 ± 5.3	79.6 ± 4.5	75.4 ± 7.8	81.8 ± 6.0	75.5 ± 5.2	ns
HR (bpm)	75.8 ± 19.8	78.7 ± 23.9	74.1 ± 10.0	72.7 ± 14.5	73.0 ± 11.8	ns
REE (kcal)	2331.3 ± 306.7	2204.0 ± 307.4	2020.8 ± 442.5	2470.1 ± 249.6	2733.0 ± 310.8	< 0.01
REE/BM (kcal/kg)	20.2 ± 3.0	19.7 ± 3.6	18.7 ± 4.6	20.8 ± 2.1	24.0 ± 2.7	< 0.01
REE/FFM (kcal/kg)	35.5 ± 5.2	34.0 ± 6.0	31.6 ± 7.2	37.2 ± 3.7	39.6 ± 2.6	ns
SCT time (sec)	3.0 ± 0.4	3.0 ± 0.4	2.8 ± 0.4	3.1 ± 0.4	2.9 ± 0.3	ns
SCT power (W)	760.9 ± 146.9	765.9 ± 163.6	785.8 ± 170.6	755.4 ± 134.1	783.3 ± 130.2	ns

BM, Body mass; BMI, Body Mass Index; BMI SDS, BMI Standard Deviation Score; FM, Fat Mass; FFM, Fat-Free Mass; SBP, Systolic Blood Pressure; DBP, Diastolic Blood Pressure; HR, Heart Rate; REE, Resting Energy Expenditure; SCT; Stair Climbing Test; ns, not significant.

*Time × Group Interaction from the two-way repeated measures ANOVA analysis.

**Table 2:** *Post-hoc* comparisons for significant time × group interactions (Bonferroni-corrected).

Outcome	Comparison	Group(s)	Adjusted p value* (bonferroni)
REE (kcal/day)	Pre vs Post (within-group)	Subgroup A	Ns
	Pre vs Post (within-group)	Subgroup B	< 0.001
	Subgroup A vs Subgroup B (between-group)	Pre	Ns
	Subgroup A vs Subgroup B (between-group)	Post	< 0.001
REE/BW (kcal/kg/day)	Pre vs Post (within-group)	Subgroup A	Ns
	Pre vs Post (within-group)	Subgroup B	< 0.001
	Subgroup A vs Subgroup B (between-group)	Pre	ns
	Subgroup A vs Subgroup B (between-group)	Post	< 0.05

BM, Body mass; REE, Resting Energy Expenditure; ns, not significant.

*Student’s t-test for paired and unpaired samples, as appropriate.

REE and anaerobic power were designated as primary outcomes; anthropometric, cardiovascular, and body composition measures were considered exploratory.

The primary outcome REE, showed a significant Time × Group interaction (p < 0.01), indicating differential responses between the two intervention groups. WBV training significantly increased REE in Subgroup B, with values rising from 2470.1 ± 249.6 kcal/day at baseline to 2733.0 ± 310.8 kcal/day post-intervention (Δ = +262.9 kcal, p < 0.001). In contrast, Subgroup A showed no significant change in REE, with values decreasing from 2204.0 ± 307.4 kcal/day to 2020.8 ± 442.5 kcal/day (Δ = ˗183.2 kcal, p = ns). Therefore, the mean difference in REE change between Subgroup B and Subgroup A was 446.1 kcal/day. Additionally, post-intervention between-group comparison revealed significantly higher REE in the WBV group compared to the control group (p < 0.001).

When REE was normalized to body mass (REE/BM), the Time × Group interaction remained significant (p < 0.01). Subgroup B demonstrated a substantial increase in weight-adjusted REE from 20.8 ± 2.1 kcal/kg/day to 24.0 ± 2.7 kcal/kg/day (p < 0.001), while Subgroup A showed no significant change (19.7 ± 3.6 to 18.7 ± 4.6 kcal/kg/day, p = ns). Post-intervention comparison between groups showed significantly higher REE/BM in the WBV group (p < 0.05). REE normalized to fat-free mass (REE/FFM) showed a significant group effect (F = 5.8, p < 0.05, partial η² = 0.27) but no significant Time × Group interaction (p = ns).

Both groups achieved significant improvements in anthropometric parameters following the 3-week BWRP. BM decreased significantly in both groups (Time effect: F = 99.43, p < 0.001, partial η² = 0.83), with Subgroup A reducing from 115.3 ± 25.5 kg to 110.2 ± 24.0 kg and Subgroup B from 119.6 ± 15.8 kg to 115.3 ± 14.9 kg. No significant Time × Group interaction was observed (p = ns), indicating similar weight loss between groups.

BMI reductions were also significant in both groups (Time effect: F = 124.98, p < 0.001, partial η² = 0.86), with Subgroup A decreasing from 38.7 ± 6.1 to 37.0 ± 5.8 kg/m² and Subgroup B from 38.3 ± 5.2 to 36.9 ± 5.4 kg/m². Similarly, BMI SDS improved significantly (Time effect: F = 24.86, p < 0.001, partial η² = 0.54) in both groups without between-group differences.

Body composition analysis revealed significant fat mass reduction (Time effect: F = 10.97, p < 0.01, partial η² = 0.41) in both groups, with Subgroup A reducing from 49.4 ± 15.5 kg to 44.0 ± 13.2 kg and Subgroup B from 50.0 ± 10.5 kg to 44.4 ± 8.6 kg. Fat-free mass percentage showed non-significant increases in both groups, suggesting preservation of lean body mass during weight loss.

Significant improvements in blood pressure were observed following the intervention in both groups. SBP decreased substantially (Time effect: F = 24.63, p < 0.001, partial η² = 0.54), with Subgroup A reducing from 128.3 ± 7.5 to 117.1 ± 10.1 mmHg and Subgroup B from 130.9 ± 13.8 to 117.3 ± 6.5 mmHg. DBP also improved significantly (Time effect: F = 14.67, p < 0.01, partial η² = 0.41), decreasing from 79.6 ± 4.5 to 75.4 ± 7.8 mmHg in Subgroup A and from 81.8 ± 6.0 to 75.5 ± 5.2 mmHg in Subgroup B. No significant Time × Group interactions were found for cardiovascular parameters, indicating similar improvements in both groups.

Resting heart rate remained stable throughout the intervention period in both groups, with no significant changes observed.

Anaerobic performance improved significantly in both groups as assessed by the Stair Climbing Test. T_SCT_ decreased significantly (Time effect: F = 70.29, p < 0.001, partial η² = 0.77), with Subgroup A improving from 3.0 ± 0.4 to 2.8 ± 0.4 seconds and Subgroup B from 3.1 ± 0.4 to 2.9 ± 0.3 seconds. Correspondingly, P_SCT_ increased significantly (Time effect: F = 12.46, p < 0.01, partial η² = 0.37) in both groups, with Subgroup A increasing from 765.9 ± 163.6 to 785.8 ± 170.6 W and Subgroup B from 755.4 ± 134.1 to 783.3 ± 130.2 W. No significant between-group differences were observed for anaerobic performance measures.

**Figure 1:**
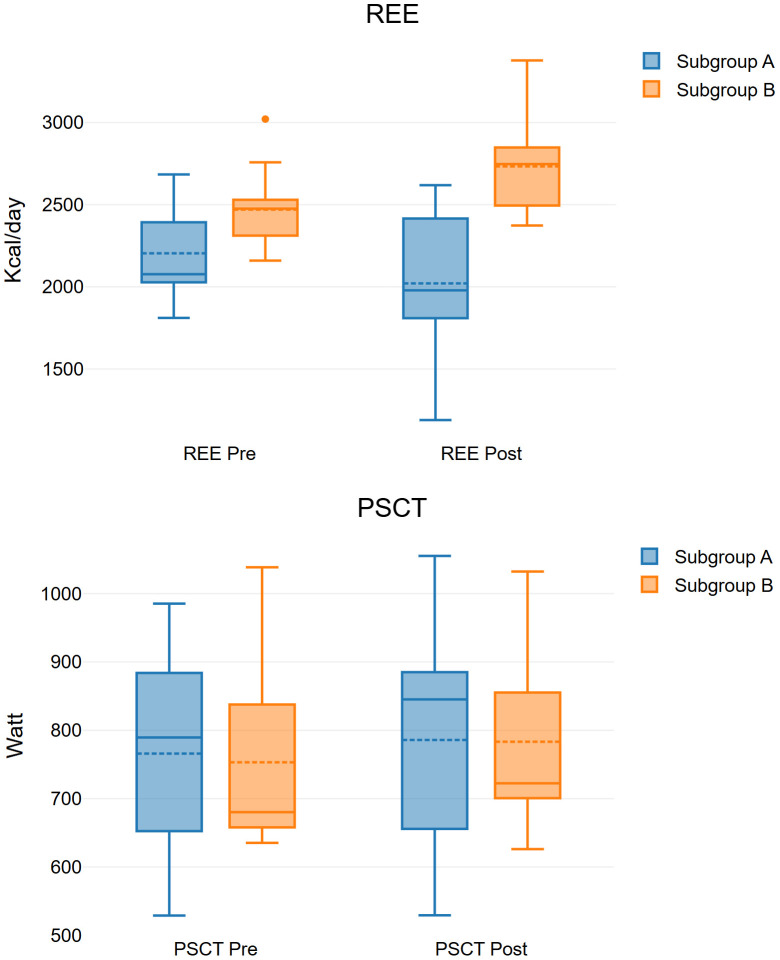
Box plot representing the change in REE and SCT power of each group.

**Figure 1** shows the change in REE and SCT power of each group.

**Table 3** shows the correlation between REE and P_SCT_ in the two subgroups, at baseline and after the intervention.

**Table 3:** Correlation between REE and PSCT in the two subgroups, at baseline and post-intervention.

	PRE		POST	
	Subgroup A	Subgroup B	Subgroup A	Subgroup B
r	0.53	0.69	0.30	0.76
r2	0.28	0.47	0.09	0.57
p value	ns	< 0.05	Ns	< 0.01
Fisher r-to-z				
z	-0.53		-1.41	
95% CI	-0.79 – 0.46		-1.11 – 0.19	
p	ns		ns	

REE, Resting Energy Expenditure; P_SCT_, Power Stair Climbing Test; ns, not significant; CI, confidence interval.

In Subgroup B, baseline REE and P_SCT_ were moderately correlated (r = 0.69, r² = 0.47). After the 3-week BWRP + WBV intervention, this relationship became more evident (r = 0.76, r² = 0.57). In the control group (Subgroup A), the baseline correlation was also moderate, although not statistically significant (r = 0.53, r² = 0.28). However, following the intervention, this correlation weakened further, resulting in a non-significant correlation (r = 0.30, r² = 0.09). However, Fisher’s r-to-z transformation revealed no statistically significant differences in correlation strength between groups at either time point.

## 4 Discussion

Several studies have evaluated the positive effects of WBV in individuals with obesity (17). In particular, WBV interventions were effective in reducing body weight and fat mass, lowering blood pressure and arterial stiffness, improving insulin sensitivity, enhancing muscle strength, and promoting fat oxidation, thereby supporting its potential role in promoting cardiovascular health (47–53). However, data regarding WBV in pediatric populations remain scarce.

To the best of our knowledge, this randomized controlled trial is the first study that evaluated the impact of adding a series of WBV training sessions to a multidisciplinary BWRP on REE in obese adolescents, compared to BWRP alone.

The current study demonstrated that including WBV in a 3-week in-hospital multidisciplinary BWRP significantly increased REE and weight-adjusted REE in obese adolescents, while maintaining comparable anthropometric improvements, cardiovascular benefits, and anaerobic performance gains compared to BWRP alone. These findings highlight the potential of WBV as an effective adjunct to conventional weight‐loss interventions in pediatric obesity.

The most significant findings of our study indicate that WBV induced a significant increase in absolute REE (approx. 10%) as well as in REE normalized to body mass (approx. 15%). A two-way repeated measures ANOVA confirmed a significant group × time interaction for REE, supporting that the observed increase in REE and REE/BM was specific to the WBV group. In fact, WBV training resulted in an average increase of approximately +263 kcal/day, while the control group showed a decline of approximately −183 kcal/day. This corresponds to an absolute between-group difference of more than 440 kcal/day, representing a clinically meaningful enhancement in resting metabolism beyond the effects of the standard multidisciplinary approach.

The divergent REE responses between groups highlight the clinical importance of addressing metabolic adaptation during weight loss interventions. Caloric restriction typically induces a reduction in energy expenditure that exceeds what would be expected from tissue losses alone, representing an adaptive mechanism that can impede long-term weight management. Our control group’s REE decline is consistent with this well-documented phenomenon, where metabolic adaptation occurs as a protective response to sustained energy restriction (54, 55). Notably, the ability of WBV to not only prevent but actually increase REE during concurrent caloric restriction may contribute to establishing and maintaining the negative energy balance necessary for sustained weight loss, potentially addressing one of the most significant barriers to long-term weight management success in adolescents with obesity.

This increase in REE aligns with prior observations in adult populations. For instance, Da Silva et al. reported that resistance exercise combined with WBV significantly increased oxygen uptake and energy expenditure compared to resistance training without vibration in active men, suggesting a role for WBV in supporting negative energy balance and preserving lean mass (32). Similarly, Rittweger et al. observed that WBV increased energy metabolic rate and glucose oxidation, potentially accelerating fat loss (33, 56). Maciejczyk et al. also showed that WBV acutely elevates REE, although the effect may be transient following a single session. Interestingly, their more recent study suggested that localized and repeated mechanical vibration applied over two weeks was capable of increasing basal oxygen consumption and REE in healthy men, highlighting a potential therapeutic application for metabolic disorders, including obesity (30, 57). Huang et al. further corroborated these findings by demonstrating increased energy expenditure and post-exercise oxygen consumption during WBV-enhanced training in trained male students (34).

By extending these observations to adolescents, the current study contributes novel evidence that WBV can acutely increase REE in the pediatric obese population, an effect that may help mitigate the metabolic adaptations often associated with caloric restriction and weight loss.

Beyond the primary metabolic outcomes, both groups achieved substantial and comparable improvements in anthropometric measures, with significant reductions in BM, BMI, and fat mass. The preservation of these benefits while simultaneously increasing REE in the WBV group suggests that vibration training provides additional metabolic advantages without compromising the effectiveness of conventional weight management approaches.

Regarding cardiovascular parameters, significant reductions in both SBP and DBP were observed in both subgroups, which are likely attributable to the overall effects of caloric restriction and exercise included in the BWRP. Importantly, no group × time interactions were observed, indicating that WBV did not induce additional hemodynamic benefits beyond those of the multidisciplinary program. While some previous studies have highlighted that WBV may cause improvement in circulation and a reduction in arterial stiffness in various populations (58–62), our findings suggest that, at least within a 3-week intervention, blood pressure improvements in obese adolescents are predominantly attributable to the core components of the BWRP rather than the WBV stimulus.

With respect to lower-limb muscle performance, both subgroups significantly improved their T_SCT_ and P_SCT_ across the intervention. However, no significant group × time interaction was observed, indicating that the multidisciplinary approach effectively enhances functional capacity regardless of WBV supplementation. Although WBV has been reported to be able to elicit neuromuscular adaptations, such as enhanced motor unit recruitment and muscle spindle sensitivity, that translate into greater explosive strength (59, 60), our findings do not support an additional effect of WBV on anaerobic power in obese adolescents. From a clinical point of view, improved lower-limb power can enable adolescents with obesity to perform common daily activities (such as stair climbing or brisk walking) with more skill, thereby enhancing their self-esteem and quality of life. Therefore, the improvement in T_SCT_ and P_SCT_ seen in both groups suggests that overall lower-limb function benefited from the structured physical activity program. At the same time the potential additive role of WBV in neuromuscular performance remains an open question for future research.

The correlation analyses provided additional insights into the interplay between metabolism and neuromuscular function. In the WBV group, the baseline association between REE and SCT power was moderate and strengthened post−intervention. By contrast, the control group also exhibited a moderate, but non-significant, baseline correlation that weakened post-intervention, suggesting that standard BWRP alone was insufficient to reinforce this metabolic–neuromuscular association. However, Fisher’s r-to-z transformation revealed no statistically significant differences in correlation strength between groups. These results suggest that REE and anaerobic performance may be associated in obese adolescents subjected to a WBV training, but such associations must be interpreted cautiously and cannot be considered causal.

Physiologically, the strong REE–power coupling observed in the WBV group may suggest an interaction between metabolic and functional adaptations. However, since FFM did not change, there is no direct evidence for increased metabolically active muscle mass specifically attributable to WBV. Likewise, while WBV has been proposed to enhance neural activation through mechanisms such as motor unit recruitment and muscle spindle sensitivity (18, 48), our study does not allow us to confirm such adaptations. By contrast, the weaker post-treatment coupling recorded in the control group may be attributable to adaptive thermogenesis and a down−regulation of resting energy expenditure during caloric restriction when relying solely on conventional exercise and diet (63, 64). Future studies with larger samples and direct assessments of neuromuscular activation are needed to clarify these potential pathways.

The current study has some limitations. The relatively short treatment period (three weeks), which, however, represents the duration of our multidisciplinary in-hospital BWRP, may not have been sufficient to detect meaningful changes in body composition, particularly in terms of FFM, or to evaluate the long-term effects of the intervention. Future studies should include longer follow‐up to assess whether WBV‐induced increases in REE might translate into greater fat mass reduction and sustained weight loss. Furthermore, the exclusive enrollment of male adolescents limits the generalizability of our findings to female adolescents, who may exhibit different metabolic or neuromuscular responses to WBV due to hormonal and physiological differences. The use of BIA for body composition assessment constitutes a limitation, especially in obese populations, where the BIA accuracy can be compromised by altered hydration status and body shape. Lastly, the relatively small sample size may reduce the statistical power to detect subtle effects and limit the broader applicability of the results. Future research with larger cohorts and alternative body composition methods, such as dual-energy X-ray absorptiometry (DEXA), is warranted to confirm and extend our findings.

Furthermore, the WBV group underwent 24 additional vibration sessions (20 minutes per day) beyond the five standard exercise sessions per week. This additional activity volume may have independently influenced the metabolic outcome. Therefore, we cannot exclude that increased total exercise volume, rather than vibration alone, contributed to the beneficial effects observed. Future studies should include an exercise‐volume–matched control to isolate the effect of mechanical vibration.

Nonetheless, the strengths of our study include its randomized, controlled design, the use of a well-characterized control group undergoing an established multidisciplinary BWRP, the use of the gold-standard indirect calorimetry for measuring REE, and a homogeneous sample of male adolescents, ensuring consistency of data. Moreover, the uniformity of our sample, comprised solely of male adolescents, reduces biological variability and strengthens the internal consistency of the results. The in-hospital setting further enhanced adherence to the protocol for both dietary and physical activity components, as well as for the WBV intervention.

In conclusion, integrating WBV into an in-hospital multidisciplinary BWRP appears to be a promising and clinically meaningful strategy for managing pediatric obesity. WBV is a safe, time-efficient, low-impact and well-tolerated type of exercise, making it particularly suitable for individuals with low exercise tolerance or mobility limitations, such as the obese population. By increasing REE, counteracting metabolic adaptations, WBV may serve as a valuable adjunct to conventional dietary and exercise interventions. Further research should investigate the positive effects of WBV on body composition and weight-loss maintenance, explore sex-specific responses, determine optimal vibration parameters, and define its long-term effects.

## Data Availability

The datasets presented in this study can be found in online repositories. The names of the repository/repositories and accession number(s) can be found below: www.zenodo.org.
