# Supplementary material for: Whole-body vibration administered during a 3-week in-hospital multidisciplinary body weight reduction program increases resting energy expenditure in obese adolescents, a randomized clinical trial
Source: Front Endocrinol (Lausanne). 2025 Sep 17;16:1642437. doi: 10.3389/fendo.2025.1642437 (PMC12483858; doi:10.3389/fendo.2025.1642437)
Supplement: Supplementary file 1 [file Table1.pdf]

Supplementary Material

Supplementary Table 1. Detailed results of the two-way repeated measures ANOVA

	F	df	p-value	Partial η² (effect size)
**BM**				
Time (BW pre vs BW post)	99.43	1, 21	< 0.001	0.83
Group	0.27	1, 21	ns	0.01
Time × Group Interaction	1.48	1, 21	ns	0.07
**BMI**				
Time (BMI pre vs BMI post)	124.98	1, 21	< 0.001	0.86
Group	0.01	1, 21	ns	0
Time × Group Interaction	3.87	1, 21	ns	0.16
**BMI SDS**				
Time (BMI SDS pre vs BMI SDS post)	24.86	1, 21	< 0.001	0.54
Group	0.05	1, 21	ns	0
Time × Group Interaction	0.39	1, 21	ns	0.02
**FFM**				
Time (FFM pre vs FFM post)	0.29	1, 16	ns	0.02
Group	0.45	1, 16	ns	0.03
Time × Group Interaction	0.55	1, 16	ns	0.03
**FM**				
Time (FM pre vs FM post)	10.97	1, 16	< 0.01	0.41
Group	0	1, 16	ns	0
Time × Group Interaction	0.47	1, 16	ns	0.03
**SBP**				
Time (SBP pre vs SBP post)	24.63	1, 21	< 0.001	0.54
Group	0.18	1, 21	ns	0.01
Time × Group Interaction	0.23	1, 21	ns	0.01
**DBP**				
Time (DBP pre vs DBP post)	14.67	1, 21	< 0.01	0.41
Group	0.29	1, 21	ns	0.01
Time × Group Interaction	0.65	1, 21	ns	0.03
**HR**				
Time (HR pre vs HR post)	0.4	1, 21	ns	0.02
Group	0.38	1, 21	ns	0.02
Time × Group Interaction	0.46	1, 21	ns	0.02
**REE**				
Time (REE pre vs REE post)	0.22	1, 21	ns	0.01
Group	15.16	1, 21	< 0.01	0.42
Time × Group Interaction	12.27	1, 21	< 0.01	0.37
**REE/BM**				
Time (REE/BM pre vs REE/BM post)	2	1, 21	ns	0.09
Group	6.32	1, 21	< 0.05	0.23
Time × Group Interaction	8.83	1, 21	< 0.01	0.3
**REE/FFM kg**				
Time (REE/FFM kg pre vs REE/FFM kg post)	0.17	1, 16	ns	0.01
Group	5.8	1, 16	< 0.05	0.27
Time × Group Interaction	2.32	1, 16	ns	0.13
**T_{SCT}**				

Time (T_{SCT} pre vs T_{SCT} post)	70.29	1, 21	< 0.001	0.77
Group	0.99	1, 21	ns	0.04
Time × Group Interaction	0.48	1, 21	ns	0.02
**P_{SCT}**				
Time (P_{SCT} pre vs P_{SCT} post)	12.46	1, 21	< 0.01	0.37
Group	0.01	1, 21	ns	0.00
Time × Group Interaction	0.52	1, 21	ns	0.02
**Abbreviations**: BM, Body mass; BMI, Body Mass Index; BMI SDS, BMI Standard Deviation Score; FM, Fat Mass; FFM, Fat Free Mass; SBP, Systolic Blood Pressure; DBP, Diastolic Blood Pressure; HR, Heart Rate; REE, Resting Energy Expenditure; SCT; Stair Climbing Test; ns, not significant.				
